# Supplementary figures and images for: Mapping Enzymatic Catalysis Using the Effective Fragment Molecular Orbital Method: Towards all ab initio Biochemistry
Source: PLoS One. 2013 Apr 12;8(4):e60602. doi: 10.1371/journal.pone.0060602 (PMC3625203; doi:10.1371/journal.pone.0060602)

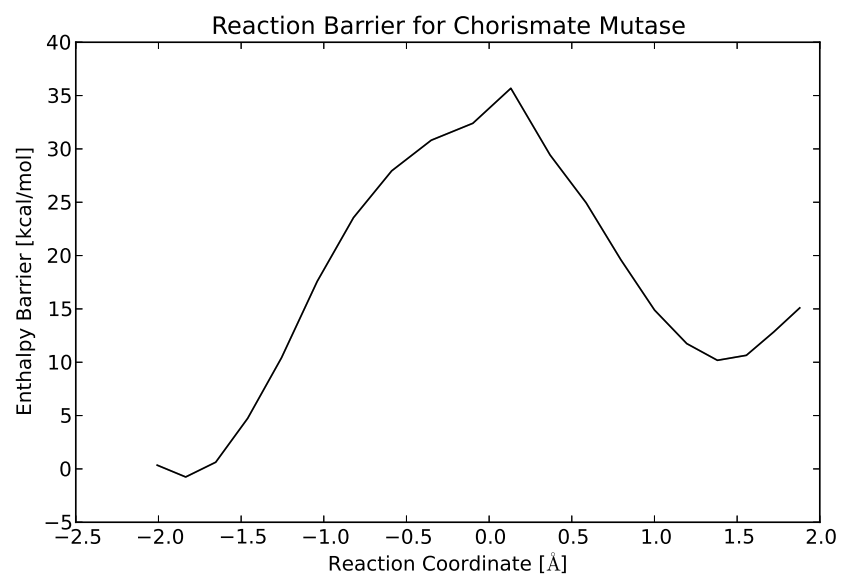

Supplement: Figure S1 — Reaction barrier calculated at the MP2/cc-pVDZ:EFMO-RHF/6-31G(d) level of theory for EFMO:S using and FDD (modfd = 3). This snapshot shows the effect of not having enough flexibility in the active region around the substrate. (PDF) [file pone.0060602.s001.pdf]

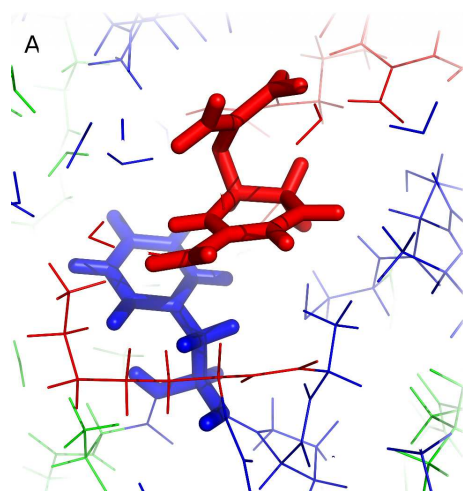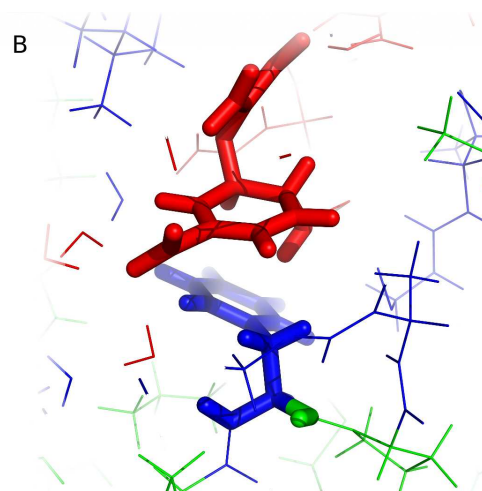

Supplement: Figure S2 — Two different starting geometries with chorismate and Phe57 shown as sticks from the MD simulation. A) shows a configuration which results in a successful reaction path and B) a configuration which results in an unsuccessful reaction path (see Figure S1). The position of Phe57 coupled with a placement in the buffer region (b) makes it unable to move to accommodate the conversion of chorismate to prephenate. (PDF) [file pone.0060602.s002.pdf]
