# Supplementary material for: Mapping Enzymatic Catalysis Using the Effective Fragment Molecular Orbital Method: Towards all ab initio Biochemistry
Source: PLoS One. 2013 Apr 12;8(4):e60602. doi: 10.1371/journal.pone.0060602 (PMC3625203; doi:10.1371/journal.pone.0060602)
Supplement: Table S1 — Complete listing of all residues in the protein model (PDB: 2CHT) along with their protonation state after being protonated using the PDB2PQR tool. (PDF) [file pone.0060602.s003.pdf]

Table S1

Residue	Chain	Charge	Residue	Chain	Charge	Residue	Chain	Charge
MET002	A	1	MET002	B	1	MET002	C	1
ILE003	A	0	ILE003	B	0	ILE003	C	0
ARG004	A	1	ARG004	B	1	ARG004	C	1
GLY005	A	0	GLY005	B	0	GLY005	C	0
ILE006	A	0	ILE006	B	0	ILE006	C	0
ARG007	A	1	ARG007	B	1	ARG007	C	1
GLY008	A	0	GLY008	B	0	GLY008	C	0
ALA009	A	0	ALA009	B	0	ALA009	C	0
THR010	A	0	THR010	B	0	THR010	C	0
THR011	A	0	THR011	B	0	THR011	C	0
VAL012	A	0	VAL012	B	0	VAL012	C	0
GLU013	A	-1	GLU013	B	-1	GLU013	C	-1
ARG014	A	1	ARG014	B	1	ARG014	C	1
ASP015	A	-1	ASP015	B	-1	ASP015	C	-1
THR016	A	0	THR016	B	0	THR016	C	0
GLU017	A	-1	GLU017	B	-1	GLU017	C	-1
GLU018	A	-1	GLU018	B	-1	GLU018	C	-1
GLU019	A	-1	GLU019	B	-1	GLU019	C	-1
ILE020	A	0	ILE020	B	0	ILE020	C	0
LEU021	A	0	LEU021	B	0	LEU021	C	0
GLN022	A	0	GLN022	B	0	GLN022	C	0
LYS023	A	1	LYS023	B	1	LYS023	C	1
THR024	A	0	THR024	B	0	THR024	C	0
LYS025	A	1	LYS025	B	1	LYS025	C	1
GLN026	A	0	GLN026	B	0	GLN026	C	0
LEU027	A	0	LEU027	B	0	LEU027	C	0
LEU028	A	0	LEU028	B	0	LEU028	C	0
GLU029	A	-1	GLU029	B	-1	GLU029	C	-1
LYS030	A	1	LYS030	B	1	LYS030	C	1
ILE031	A	0	ILE031	B	0	ILE031	C	0
ILE032	A	0	ILE032	B	0	ILE032	C	0
GLU033	A	-1	GLU033	B	-1	GLU033	C	-1
GLU034	A	-1	GLU034	B	-1	GLU034	C	-1
ASN035	A	0	ASN035	B	0	ASN035	C	0
HIS036	A	0	HIS036	B	0	HIS036	C	0
THR037	A	0	THR037	B	0	THR037	C	0
LYS038	A	1	LYS038	B	1	LYS038	C	1
PRO039	A	0	PRO039	B	0	PRO039	C	0
GLU040	A	-1	GLU040	B	-1	GLU040	C	-1
ASP041	A	-1	ASP041	B	-1	ASP041	C	-1
VAL042	A	0	VAL042	B	0	VAL042	C	0
VAL043	A	0	VAL043	B	0	VAL043	C	0
GLN044	A	0	GLN044	B	0	GLN044	C	0
MET045	A	0	MET045	B	0	MET045	C	0
LEU046	A	0	LEU046	B	0	LEU046	C	0

Continued on next page

Table S1 – continued from previous page

Residue	Chain	Charge	Residue	Chain	Charge	Residue	Chain	Charge
LEU047	A	0	LEU047	B	0	LEU047	C	0
SER048	A	0	SER048	B	0	SER048	C	0
ALA049	A	0	ALA049	B	0	ALA049	C	0
THR050	A	0	THR050	B	0	THR050	C	0
PRO051	A	0	PRO051	B	0	PRO051	C	0
ASP052	A	-1	ASP052	B	-1	ASP052	C	-1
LEU053	A	0	LEU053	B	0	LEU053	C	0
HIS054	A	0	HIS054	B	0	HIS054	C	0
ALA055	A	0	ALA055	B	0	ALA055	C	0
VAL056	A	0	VAL056	B	0	VAL056	C	0
PHE057	A	0	PHE057	B	0	PHE057	C	0
PRO058	A	0	PRO058	B	0	PRO058	C	0
ALA059	A	0	ALA059	B	0	ALA059	C	0
LYS060	A	1	LYS060	B	1	LYS060	C	1
ALA061	A	0	ALA061	B	0	ALA061	C	0
VAL062	A	0	VAL062	B	0	VAL062	C	0
ARG063	A	1	ARG063	B	1	ARG063	C	1
GLU064	A	-1	GLU064	B	-1	GLU064	C	-1
LEU065	A	0	LEU065	B	0	LEU065	C	0
SER066	A	0	SER066	B	0	SER066	C	0
GLY067	A	0	GLY067	B	0	GLY067	C	0
TRP068	A	0	TRP068	B	0	TRP068	C	0
GLN069	A	0	GLN069	B	0	GLN069	C	0
TYR070	A	0	TYR070	B	0	TYR070	C	0
VAL071	A	0	VAL071	B	0	VAL071	C	0
PRO072	A	0	PRO072	B	0	PRO072	C	0
VAL073	A	0	VAL073	B	0	VAL073	C	0
THR074	A	0	THR074	B	0	THR074	C	0
CYS075	A	0	CYS075	B	0	CYS075	C	0
MET076	A	0	MET076	B	0	MET076	C	0
GLN077	A	0	GLN077	B	0	GLN077	C	0
GLU078	A	-1	GLU078	B	-1	GLU078	C	-1
MET079	A	0	MET079	B	0	MET079	C	0
ASP080	A	-1	ASP080	B	-1	ASP080	C	-1
VAL081	A	0	VAL081	B	0	VAL081	C	0
THR082	A	0	THR082	B	0	THR082	C	0
GLY083	A	0	GLY083	B	0	GLY083	C	0
GLY084	A	0	GLY084	B	0	GLY084	C	0
LEU085	A	0	LEU085	B	0	LEU085	C	0
LYS086	A	1	LYS086	B	1	LYS086	C	1
LYS087	A	1	LYS087	B	1	LYS087	C	1
CYS088	A	0	CYS088	B	0	CYS088	C	0
ILE089	A	0	ILE089	B	0	ILE089	C	0
ARG090	A	1	ARG090	B	1	ARG090	C	1
VAL091	A	0	VAL091	B	0	VAL091	C	0
MET092	A	0	MET092	B	0	MET092	C	0
MET093	A	0	MET093	B	0	MET093	C	0

Continued on next page

Table S1 – continued from previous page

Residue	Chain	Charge	Residue	Chain	Charge	Residue	Chain	Charge
THR094	A	0	THR094	B	0	THR094	C	0
VAL095	A	0	VAL095	B	0	VAL095	C	0
GLN096	A	0	GLN096	B	0	GLN096	C	0
THR097	A	0	THR097	B	0	THR097	C	0
ASP098	A	-1	ASP098	B	-1	ASP098	C	-1
VAL099	A	0	VAL099	B	0	VAL099	C	0
PRO100	A	0	PRO100	B	0	PRO100	C	0
GLN101	A	0	GLN101	B	0	GLN101	C	0
ASP102	A	-1	ASP102	B	-1	ASP102	C	-1
GLN103	A	0	GLN103	B	0	GLN103	C	0
ILE104	A	0	ILE104	B	0	ILE104	C	0
ARG105	A	1	ARG105	B	1	ARG105	C	1
HIS106	A	0	HIS106	B	0	HIS106	C	0
VAL107	A	0	VAL107	B	0	VAL107	C	0
TYR108	A	0	TYR108	B	0	TYR108	C	0
LEU109	A	0	LEU109	B	0	LEU109	C	0
GLU110	A	-1	GLU110	B	-1	GLU110	C	-1
LYS111	A	1	LYS111	B	1	LYS111	C	1
ALA112	A	0	ALA112	B	0	ALA112	C	0
VAL113	A	0	VAL113	B	0	VAL113	C	0
VAL114	A	0	VAL114	B	0	VAL114	C	0
LEU115	A	-1	LEU115	B	0	LEU115	C	0
			ARG116	B	1	ARG116	C	1
			PRO117	B	0	PRO117	C	0
			ASP118	B	-1	ASP118	C	-2
			LEU119	B	-1			
