# Supplementary material for: Mapping Enzymatic Catalysis Using the Effective Fragment Molecular Orbital Method: Towards all ab initio Biochemistry
Source: PLoS One. 2013 Apr 12;8(4):e60602. doi: 10.1371/journal.pone.0060602 (PMC3625203; doi:10.1371/journal.pone.0060602)
Supplement: Table S4 — Reaction barriers of chorismate mutase calculated using ONIOM for EFMO:L using and FDD (modfd = 3). (PDF) [file pone.0060602.s006.pdf]

Table S4.

	R_R	R_{TS}	R_P	E_{TS-R}	E_{P-R}
MP2/6-31G(d)	-1.83	-0.11	1.57	22.15	-5.69
MP2/cc-pVDZ	-1.83	-0.35	1.57	19.92	-7.97
B3LYP/6-31G(d)	-1.83	0.13	1.57	24.90	1.35
B3LYP/cc-pVDZ	-1.83	0.13	1.57	23.50	0.12

The reaction coordinates in Å for the reactant, transition state and product are R_R, R_{TS} and R_P, respectively. The barrier height of the transition state $E_{\mathrm{TS-R}}$ and the overall reaction energy $E_{\mathrm{P-R}}$ are in kcal/mol.
